# Brazilian registry of patients with porphyria: REBRAPPO study

**DOI:** 10.1186/s13023-023-02653-1

**Published:** 2023-03-08

**Authors:** Paulo Victor Sgobbi Souza, Gliciane Afonso, Wladimir Bocca Vieira de Rezende Pinto, Paulo de Lima Serrano, Bruno de Mattos Lombardi Badia, Igor Braga Farias, Ana Carolina dos Santos Jorge, Roberta Ismael Lacerda Machado, Icaro França Navarro Pinto, Glenda Barbosa Barros, Helvia Bertoldo de Oliveira, Samia Rogatis Calil, Cibele Franz, Acary Souza Bulle Oliveira

**Affiliations:** 1 Neuromuscular Unit, Department of Neurology and Neurosurgery, Federal University of São Paulo (UNIFESP), Embaú Street, 67, Vila Clementino, São Paulo, SP 04039-060 Brazil; 2 Universidade Anhanguera, Campinas, Brazil; 3 Universidade Federal do Estado do Rio de Janeiro, Rio de Janeiro, Brazil

**Keywords:** Porphyrias, Acute hepatic porphyria, Heme metabolism, Hereditary rare diseases, Givosiran, Natural history, Porphobilinogen, Aminolevulinic acid

## Abstract

**Background:**

Porphyrias are a rare group of disease due to inherited defects of heme synthesis with important systemic manifestations and great burden of disease for patients and families due to the exceptional course of disease with disabling chronic symptoms interposed by life-threatening acute attacks. Unfortunately, the porphyrias are usually underrecognized reflecting a lack of medical and disease awareness as well as few studies about natural history in large cohorts of patients. The main aim of this article is present consistent data about natural history and burden of disease in a large Brazilian cohort.

**Methods:**

We conducted a national cross-sectional registry with retrospective clinical data of Brazilian patients with porphyria collected with Brazilian patients Association with Porphyria in collaboration with a tertiary care center for rare diseases.

**Results:**

A cohort of 172 patients was analyzed in which 148 (86%) patients had the diagnosis of acute hepatic porphyria [AHP] that needed a mean of 62.04 medical visits and 9.6 years to achieve a definitive diagnosis. About AHP cohort, the most common first clinical manifestation were abdominal pain in 77 (52%) patients and acute muscle weakness in 23 (15.5%) with 73 (49.3%) patients presenting only one attack during disease course and 37 (25%) exhibiting 4 or more attacks in the last year. Of note, 105 patients with AHP reported chronic manifestations and the scores for quality of life are lower when compared with general healthy population.

**Conclusions:**

Brazilian patients with AHP had a higher prevalence of chronic disabling manifestations and a poor quality of life like other cohorts and a higher proportion of patients with recurrent attacks than previously reported.

## Background

Heme group production occurs predominantly in erythroblasts from the bone marrow (almost 80% of total heme production) and liver (around 20% of total heme group) and plays a major role as cofactor of different iron-based hemeproteins, such as hemoglobin, hepatic cytochromes P450, myoglobin, mitochondrial respiratory chain cytochromes and special enzymes (catalase, peroxidase, tryptophan pyrrolase, and nitric oxide synthase) [1, 2].

Porphyrias (from the Greek *Porphyrus*, purple) are a rare group of inherited or acquired metabolic disorders of the heme biosynthesis pathway and can be classified as either *erythropoietic* or *hepatic*, depending on the principal site of accumulation of pathway intermediates and based on clinical manifestations as acute porphyrias characterized by neurovisceral attacks with abdominal pain and neurologic features or chronic porphyrias with prominent cutaneous involvement in photoexposed skin areas due to overproduction of photosensitizing porphyrins [2, 3].

The first biochemical reaction in the heme biosynthesis pathway is the rate-limiting step and differs between liver and erythropoietic tissues, mostly because of differences in the activity of the first enzyme known as 5-aminolaevulinic acid synthase (ALAS) that is encoded by two genes, the ubiquitous delta-aminolevulinic acid synthase 1 gene (*ALAS1*, located on chromosome 3) in the liver and by the erythroid tissue-specific *ALAS2* gene (located on chromosome X) in bone marrow [1–3].

The production of heme group in the liver and is controlled via negative-feedback regulation by the intracellular uncommitted heme pool and modulated by individual high-susceptibility risk factors (i.e., cytochrome P450-coding genes and their polymorphisms), endocrine (i.e., menstrual cycle, pregnancy, and puerperium), environmental (i.e., lead poisoning), dietetic factors (i.e., alcohol consumption, prolonged fasting or low carbohydrate diet, and cigarette smoking), and unsafe porphyrinogen drugs (i.e., hormonal contraceptives, phenytoin, barbiturates, and sulfonamides) [2–4].

Acute Hepatic Porphyrias (AHP) represent a rare group of four inherited metabolic disorders: Acute Intermittent Porphyria (AIP) caused by porphobilinogen deaminase enzyme deficiency due to *HMBS* genetic variants; Variegate Porphyria (VP) caused by protoporphyrinogen oxidase enzyme deficiency with *PPOX* genetic variants; Hereditary Coproporphyria (HCP) with coproporphyrinogen oxidase enzyme defect related to pathogenic variants in *CPOX* gene; and delta-aminolevulinate dehydratase (ALAD) deficiency porphyria (also known as ALAD deficiency or Doss porphyria) with abnormal enzyme activity of ALA dehydratase coded by *ALAD* gene [2–4]. AHP leads to marked overproduction of porphyrins and their precursors in the liver and abnormal accumulation of intermediate metabolites, mainly porphobilinogen (PBG) and delta-aminolevulinic acid (ALA) [2–4].

More recently, small interfering RNA (siRNA)-based therapies for AHP (Givosiran, an *N*-acetyl-D-galactosamine-conjugated siRNA that specifically targeting *ALAS1* messenger RNA in the liver) have shown important results in controlling recurrent attacks and improving the overall quality of life of patients. There are, however, few studies about the natural history of disease with consistent big data about the diagnostic journey and clinical profile in a large cohort of patients [5, 6].

This article presents a large national Brazilian cohort of patients with porphyria that aims to provide insights into the epidemiology, patient’s journey, diagnosis, clinical profile, natural history and quality of life with clinical implications for doctors, health agents and patients with porphyria that can be applied worldwide.

## Methods

The Brazilian Registry of Patients with Porphyria (REBRAPPO) is a single research registry developed by the Neuromuscular Unit of Federal University of São Paulo in collaboration with Brazilian Porphyria Association (ABRAPO) that collected retrospective self-reported medical data from Brazilian patients with porphyria through a specific electronic form developed by the authors. The inclusion period was 90 days from December 1st, 2021 to February 28th, 2022. All patients gave written informed consent for participation in the study and for this publication, and study procedures were approved by institutional ethics committees (CAAE: 50086121.4.0000.5505). This research was conducted following the principles of the Declaration of Helsinki for good practice.

The electronic survey form was based on four axes: (1) demographical data, (2) disease history and medical information, (3) treatments and interventions, and (4) quality of life evaluated by the SF-12 Health Survey. The demographical data of interest for this study was: gender, residence, ethnicity, scholarity, monthly income, health coverage and occupation.

To understand disease history and medical information the following data were collected: age at onset, age at diagnosis, time to definitive diagnosis, subtype of porphyria, type of AHP, weight, height, body mass index (BMI), number of medical specialists consulted, number of medical visits until diagnosis, medical specialist who performed the diagnosis, number of family members with the diagnosis of porphyria, clinical manifestations related to porphyria, number of attacks and number of attacks in the last year for AHP patients, number of patients with chronic symptoms and clinical profile of chronic manifestations. For this study the definition of a porphyria attack was a clinical manifestation related to disease resulting in hospitalization, medical assistance on urgent health care or intravenous administration of hemin as previously described in the ENVISION study [5]. Recurrent attacks are defined as four or more attacks per year as previously described in the literature [5–7].

The chronic clinical manifestations and symptoms related to the population with AHP were categorized into four domains: (1) pain, (2) mood and sleep, (3) gastrointestinal and (4) other manifestations, similar to the EXPLORE study [6].

The third axis about treatments and interventions consisted of the following information: opioid consumption, use of glucose solution, hemin cycles, liver transplantation and treatment with Givosiran.

The quality of life was evaluated asking for patients with porphyria to describe their health-related quality of life (HRQoL) using the SF-12 Health Survey Version 2 (SF-12v2) that is a health profile instrument with 12-items to evaluate eight sub-domains classified in physical functioning (PF), role physical (RP), bodily pain (BP), general health (GH), vitality (VT), social functioning (SF), role-emotional (RE) and mental health (MH) [8, 9]. These eight sub-domain scores can be weighted and summarized into two component scores: the physical component summary (PCS) score and the mental component summary (MCS) score [8, 9]. The reference value for PCS and MCS scores for the US population have a mean of 50 and a standard deviation of 10 with lower score indicating a poor health status [8, 9].

The inclusion criteria for the study were: (1) male or female Brazilian patients with a diagnosis of symptomatic porphyria established by genetic test or biochemical analysis (fecal, urinary or plasmatic porphyrins or 24-h urinary ALA and PBG) at any age. After a detailed review of electronic answers, data were abstracted, de-identified by the collaborating investigators at the local study site and submitted through the secure Research Electronic Data Capture (REDcap) platform [10]. Patients with incomplete or duplicated data were excluded from the final study analysis.

The study primary outcomes were: (a) establish the demographical and epidemiological profile of Brazilian patients with porphyria, (b) comprehend the diagnostic journey and natural history related to disease, (c) evaluate the treatment options available and how they are used at Brazil, (d) estimate the quality of life and burden of disease.

The secondary outcomes were: (a) create a robust database that can be used for other clinical studies and for planning public health actions, (b) develop a platform with continuous data inclusion that allows obtaining special epidemiological data such as incidence and prevalence of porphyrias in a continental-sized country and monitoring the long-term impact of new therapies on the natural history of porphyrias trough data on survival and resource utilization of health services.

Descriptive statistics was applied, and categorical variables were summarized using counts and percentages of the total population. Continuous variables were reported using mean and interquartile range (IQR).

The Shapiro–Wilk and Kolmogorov–Smirnov (with Lilliefors adjustments) tests were performed to evaluate the normal distribution of the sample, and all variables proved to fit the normal distribution by at least one method.

Correlations between age at onset, age at diagnosis, time for diagnosis, number of attacks during disease course, number of attacks in the last years, PCS and MCS were performed with Pearson correlation coefficient with a correlation coefficient of r < 0.3 considered as weak, r = 0.3–0.59 a moderate, and r ≥ 0.6 a strong correlation and two‐sided *p* < 0.05 was considered statistically significant. One-way ANOVA was performed to compare the results of PCS and MCS scores according to the ‘number of attacks in the last year’.

The software Stata® 16.0 (StataCorp, College Station, TX) was used for statistical analysis.

## Results

### Study population and demographical data

This study presents a cohort of 172 patients with porphyria that fully met the inclusion criteria of whom 147 (85.4%) are female, 96 (55.8%) are Caucasian and 82 (47.6%) patients are unmarried. The educational profile showed 69 (40.1%) patients had higher education degrees and just 4 (2.3%) had less than 5 years of education. The occupation data indicated that 35 (20.3%) patients are unemployed, 86 (50%) earn between 2 and 5 minimum wage per month and 52 (30.2%) live on less than one time minimum wage. Regarding access to health services, 100 (58.1%) patients had a private health insurance and 72 (41.8%) depend exclusively on the Brazilian public health system. Seventy-nine (45.9%) live in the southeastern region of the country and 47 (27.3%) patients of the national cohort live in São Paulo which is the most densely populated province in Brazil.

**Fig. 1:**
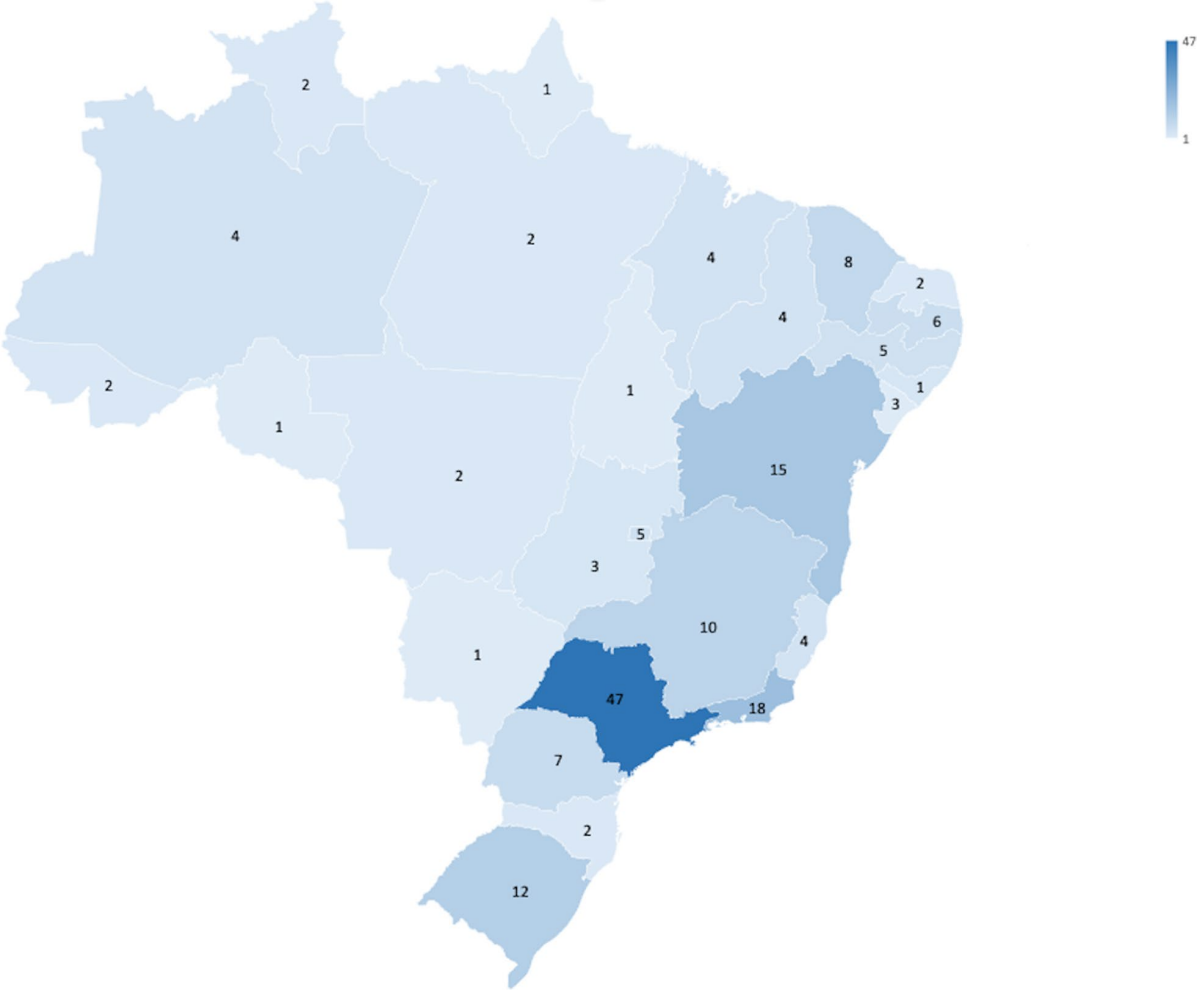
Geographical distribution of patients with Porphyria by the federal states of Brazil

All demographical data are summarized in Table 1 and the geographic distribution of patients throughout the Brazilian territory is shown in Fig. 1.

**Table 1:** Demographical Data

Demographical Data	Population (%)
*Gender*	
Male	25 (14.5%)
Female	147 (85.4%)
*Ancestry/ethnicity*	
White/Caucasian	96 (55.8%)
Black/African American	52 (30.2%)
Asian	12 (6.9%)
Hispanic	7 (4.0%)
Ashkenazi Jewish	4 (2.3%)
Sephardic Jewish	1 (0.5%)
*Marital status*	
Single/Unmarried	82 (47.6%)
Married	59 (34.3%)
Divorced	31 (18.0%)
Scholarity	
Elementary School	4 (2.3%)
High School	88 (51.1%)
Undergraduate School	69 (40.1%)
Graduate School	11 (6.3%)
*Employment*	
Unemployed	35 (20.3%)
Retired	42 (24.4%)
Formal Employment	62 (36.0%)
Informal Employment	12 (6.9%)
Student	21 (12.2%)
*Income*	
< 1 Brazilian minimum wage	52 (30.2%)
2 -5 Brazilian minimum wage	86 (50.0%)
5–10 Brazilian minimum wage	20 (11.6%)
> 10 Brazilian minimum wage	14 (8.1%)
*Health access*	
Brazilian Public Health System	72 (41.8%)
Private Health Insurance	100 (58.2%)
*Brazilian geographic distribution*	
North	13 (7.5%)
Northeast	48 (27.9%)
Midwest	11 (6.4%)
Southeast	79 (45.9%)
South	21 (12.2%)

Observation: This table should be placed at page 10 after line 15

### Disease history and medical information

In this cohort of 172 patients, 148 (86%) declare the diagnosis of AHP, 4 (2.3%) had Erythropoietic Porphyria (EP) and 20 (11.6%) were unaware of the disease subtype. Of the 4 patients with EP all had genetic and biochemical test confirmation with 1 patient exhibiting Congenital Erythropoietic Porphyria due to recessive variant on *UROS* gene, 1 had Erythropoietic Protoporphyria due to biallelic variants on *FECH* gene and 2 with Porphyria Cutanea Tarda due to *UROD* variants.

Of the 148 patients with AHP, 118 (79.7%) had AIP, 16 (10.8%) had VP, 9 (6.0%) had HCP and 2 (1.35%) presented ALAD deficiency with a total of 100 (67.5%) confirmed by genetic test.

Regarding the diagnostic journey of patients with AHP, 72 (48.6%) was diagnosed by a neurologist, 34 (22.9%) by a hematologist, 17 (11.4%) by a geneticist and only 12 (8.1%) received the diagnosis by a gastroenterologist or hepatologist. A total of 66 (44.5%) patients with AHP consulted more than five different medical specialists until the final diagnosis and 39 (26.3%) needed only one medical specialist to establish the final diagnosis. The patients with AHP needed a mean of 62.04 [IQR 5.0–50.5] medical consultations to achieve the final diagnosis, with 15 (10.1%) received the diagnosis at first medical visit and 24 (16.2%) required more than 100 medical visits to complete the diagnosis. According to family history, 54 (36.4%) patients with AHP reported no family history for porphyria and 11 (7.4%) had more than 10 family members affected by the disease. A summary of clinical data is shown in Table 2.

**Table 2:** Clinical Data Summary

Clinical characteristics	Population (%)
*Porphyria’s Subtype (N = 172)*	
Acute Hepatic Porphyria (AHP)	148 (86%)
Erythropoietic Porphyria (EP)	4 (2.3%)
Unknown	20 (11.6%)
*Diagnostic methods (N = 172)*	
Genetic Test + Biochemical Analysis	104 (60.4%)
Biochemical Test	68 (39.5%)
Acute hepatic porphyria population	
*Subtypes of Acute Hepatic Porphyria (N = 148)*	
Acute intermittent porphyria	118 (79.7%)
Variegate Porphyria	16 (10.8%)
Hereditary Coproporphyria	9 (6.0%)
ALAD Deficiency Porphyria	2 (1.35%)
*Definitive Diagnosis performed by*	
Neurologist	72 (48.6%)
Hematologist	34 (22.9%)
Geneticist	17 (11.4%)
Gastroenterologist / Hepatologist	12 (8.1%)
Pediatrician	4 (2.7%)
Rheumatologist	3 (2%)
Intensive Care / Emergency Medicine	3 (2%)
Psychiatry	2 (1.3%)
Dermatologist	1 (0.6%)
*Family History*	
Negative	54 (36.4%)
1 relative with AHP	47 (31.7%)
2–5 relatives with AHP	24 (16.2%)
5–10 relatives with AHP	12 (8.1%)
> 10 relatives with AHP	11 (7.4%)
*First Clinical Complaint*	
Abdominal Pain	77 (52%)
Acute Muscle Weakness	23 (15.5%)
Acute Encephalopathy	16 (10.8%)
Dark Urine	13 (8.7%)
Skin Abnormalities	9 (6.0%)
Acute Psychosis	8 (5.4%)
Seizures	2 (1.3%)
*Number of Attacks (life)*	
1 attack	73 (49.3%)
2 attacks	15 (10.1%)
3 attacks	7 (4.7%)
4 attacks	3 (2.0%)
5–10 attacks	21 (14.1%)
11–20 attacks	15 (10.1%)
> 20 attacks	14 (9.4%)
*Number of Attacks (last year)*	
None	70 (47.2%)
1 attack	30 (20.2%)
2–3 attacks	11 (7.4%)
4 or more attacks	37 (25%)
*Long-Term Complications*	
Chronic Symptoms	105 (70.9%)
Systemic Arterial Hypertension	52 (35.1%)
Family History for Hepatocarcinoma	8 (5.4%)
Chronic Renal Disease Stage V	5 (3.3%)

Observation: This table should be placed at page 11 after line 14

The mean age at diagnosis was 30.2 years [IQR 22.0–36.0], the mean age at symptom onset was 20.5 years [IQR 14.0–27.0], the mean age at first attack was 24.6 years [IQR 18.0–30.25] and the mean time to definitive diagnosis was 9.6 years [IQR 3.0–14.0]; of the 148 patients with AHP, 11 (7.4%) received the diagnosis at pediatric age under 18 years and 61 (41.2%) had the symptom onset started before the age of 18 years. The mean weight of patients with AHP was 72.0 kg [IQR 60.0–83.0] with 40 (27.0%) patients weighing more than 80 kg, the mean height was 1.64 m [IQR 1.58–1.68] and mean BMI was 26.67 kg/m^2^ [IQR 23.26–29.64] with 33 (22.2%) patients with BMI greater than 30 kg/m^2^.

The first clinical manifestation related to disease was abdominal pain in 77 (52.0%), acute muscle weakness in 23 (15.5%), acute encephalopathy in 16 (10.8%), dark urine in 13 (8.7%), skin abnormalities in 9 (6.0%) and acute psychosis in 8 (5.4%) of 148 patients with AHP.

The analysis of porphyria attacks showed a mean 6.2 [IQR 1.0–9.0] crisis per patient with 73 (49.3%) patients having only 1 attack and 53 (35.8%) presented with 4 or more attacks over the course of disease. In the last year, 70 (47.2%) patients reported no attacks, 30 (20.2%) had 1 attack and 37 (25%) had 4 or more attacks. For patients with recurrent attacks, the mean of attacks in the last year was 6.91 [IQR 5.0–8.0]. Chronic symptoms between attacks were reported by 105 (70.9%) patients. There is a strong correlation between the number of attacks in the last year with the number of attacks during disease course (r = 0.87).

The clinical profile during an attack for the cohort of 148 patients with AHP according to the four domains established above was: 1) pain, abdominal pain was reported by 140 (94.5%) patients, arm or leg pain by 136 (91.8%), muscle pain by 121 (81.7%) and back pain by 112 (75.6%); 2) mood and sleep complaints, fatigue occurred in 139 (93.9%) patients, anxiety in 132 (89.1%), insomnia in 125 (84.4%), trouble concentrating in 98 (66.2%) and hallucinations in 44 (29.7%); 3) gastrointestinal symptoms, nausea was present in 144 (97.2%) patients, loss of appetite in 113 (76.3%), constipation in 103 (69.5%) and diarrhea in 27 (18.2%); 4) other manifestations, palpitation was reported by 135 (91.2%) patients, numbness by 129 (87.1%), weakness by 111 (75%), dark urine by 95 (64.1%), fever by 56 (37.8%) and blisters/rashes by 19 (12.8%).

In relation to the 105 patients with AHP that reported chronic symptoms, the clinical profile was: 1) pain domain, arm or leg pain was observed in 63 (60%), abdominal pain in 51 (48.5%) and muscle pain in 48 (45.7%); 2) mood and sleep complaints, fatigue was reported by 82 (78%) patients, anxiety by 73 (69.5%), insomnia by 64 (60.9%), trouble concentrating by 53 (50.4%) and feeling unmotivated by 47 (44.7%); 3) gastrointestinal symptoms, constipation occurred in 60 (57.1%) patients, nausea in 53 (50.4%), heartburn in 38 (36.1%) and loss of appetite in 32 (30.4%); 4) other manifestations, numbness was present in 67 (63.8%) patients, weakness in 63 (60%), palpitation in 48 (45.7%), shakiness in 42 (40%), dark urine in 37 (35.2%) and blisters/rashes in 15 (14.2%). Medical history for systemic arterial hypertension was reported by 52 (35.1%) and positive family history for hepatocarcinoma was informed by 8 (5.4%) of 148 patients with AHP.

**Fig. 2:**
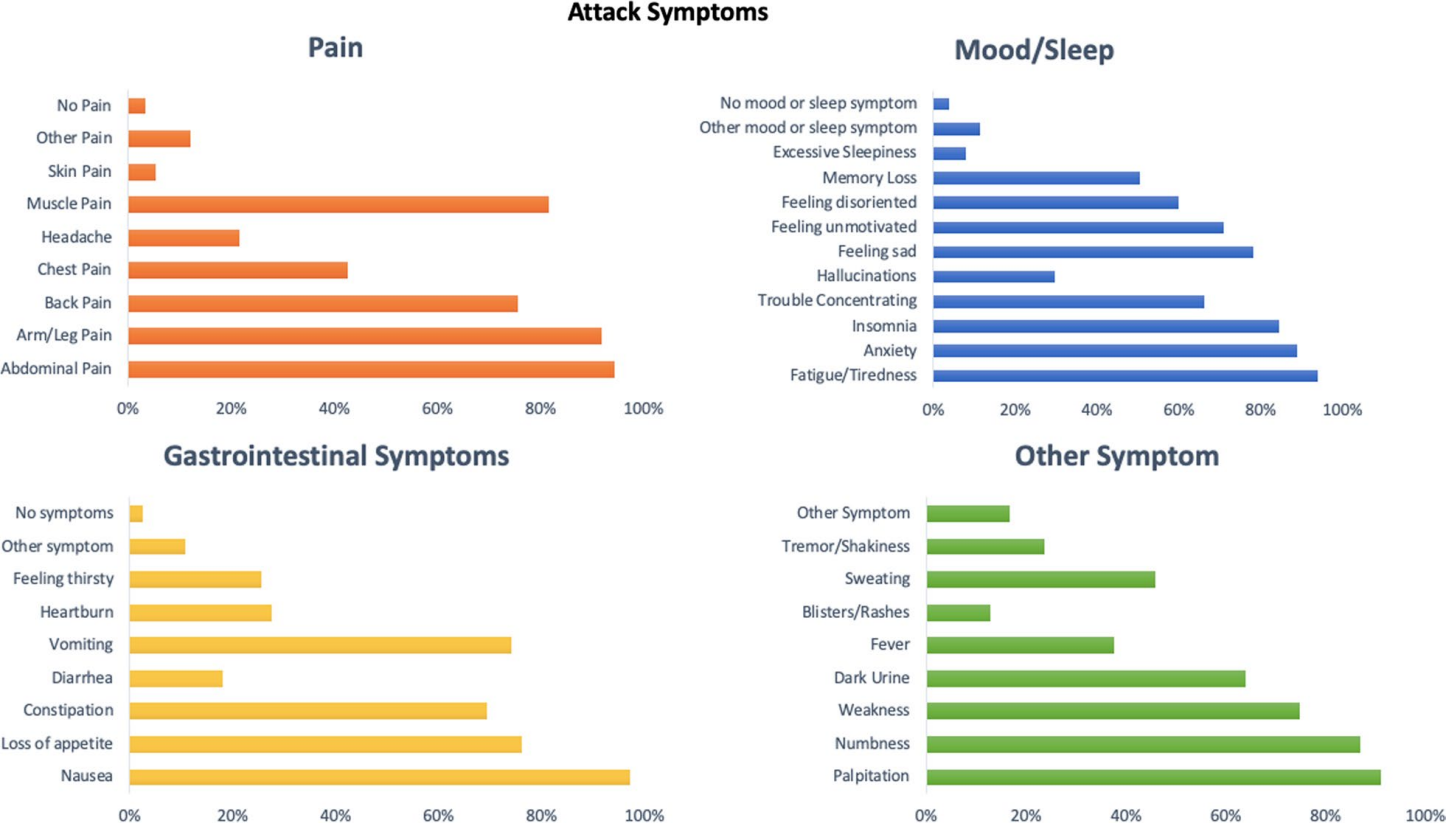
Clinical profile of acute attacks for patients with Acute Hepatic Porphyria

**Fig. 3:**
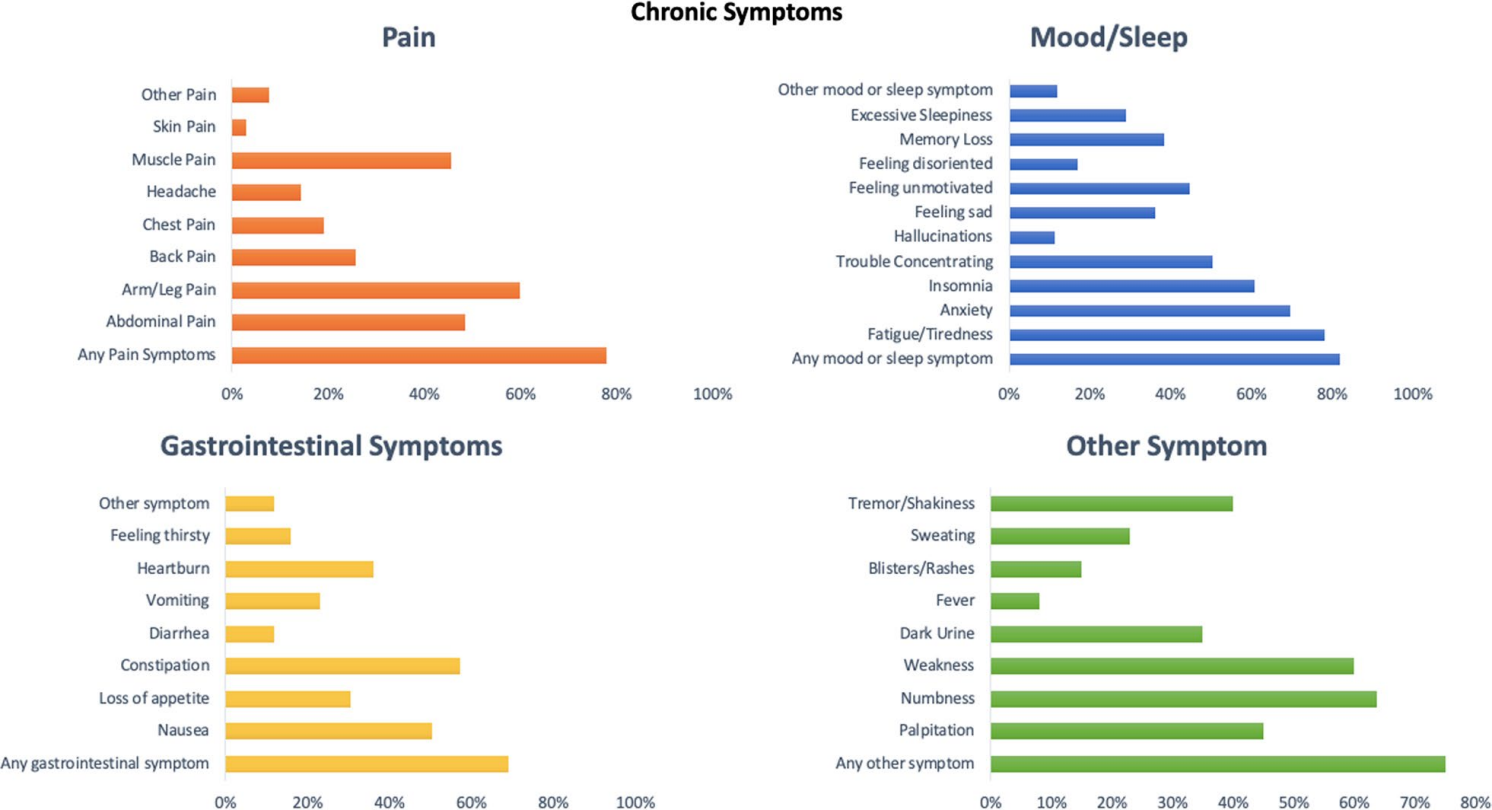
Clinical profile of chronic manifestations for patients with Acute Hepatic Porphyria

The clinical profile with percentage of patients reporting symptoms during an attack and chronic symptoms are shown on Fig. 2 and Fig. 3.

### Treatments and Interventions

From the cohort of 148 patients with AHP, 137 (92.5%) patients reported being treated with glucose solution and 57 (38.5%) informed treatment with hemin (Normosang® or Panhematin®) during at least one attack. Of the 57 patients that received hemin, 19 (33.3%) were treated on an isolated attack, 24 (42.1%) were treated in 2 to 5 different occasions, 6 (10.5%) in 6 to 10 attacks and 8 (14.0%) patients received hemin in more than 10 different attacks. The mean duration of a hemin treatment cycle was 7.6 days [IQR 5.0–10.0] and 5 (3.3%) of 148 patients with AHP are undergoing prophylactic treatment with hemin monthly.

Only 1 patient underwent live transplantation and 2 patients are being treated with Givosiran (Givlaari®). Regarding pain treatment, 109 (73.6%) of 148 patients with AHP informed opioid use in at least one attack and 47 (31.7%) reported daily use of opioids for pain relief.

### Quality of life

The mean PCS was 39.49 [IQR 30.84–47,14] and the mean MCS was found to be 35.48 [IQR 28.48–41.06] for the cohort of AHP patients. Mean PCS and MCS scores were lower than the norm scores for the general healthy US population and this indicated that Brazilian patients with AHP had a worse overall HRQoL as compared to the US norm population.

It is noteworthy that 51 (34.4%) of the 148 patients with AHP indicated that their general health is ‘fair’ or ‘poor’. During the past week, 66 (44.5%) patients declared that pain interfered with work ‘quite a bit’ or ‘extremely’ and just 3 (2%) pointed have a lot of energy ‘all of the time’. The PCS had a moderate inverse correlation with the ‘number of attacks in the last year’ (r = − 0.50) and MCS scores did not correlate with ‘time from onset of disease to final diagnosis’, ‘number of attacks in the last year’ or with ‘number of attacks during the disease course’.

**Fig. 4:**
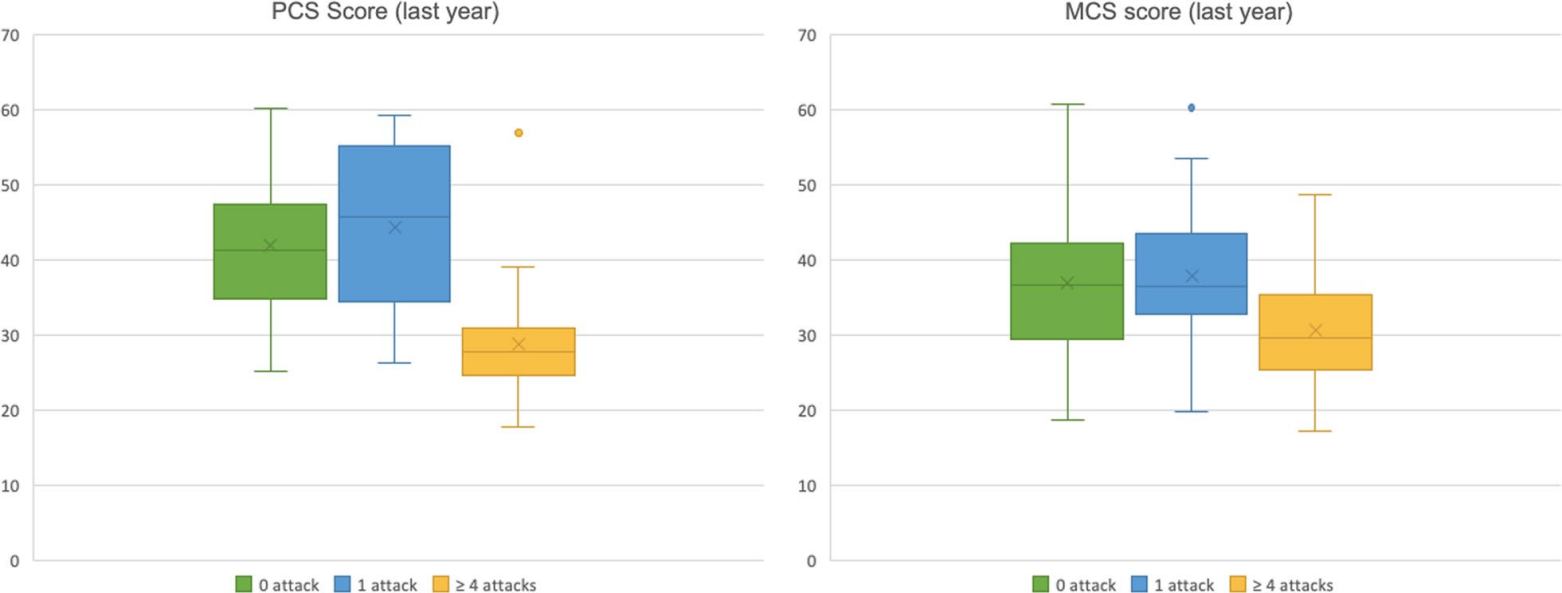
The quality-of-life profile with Physical Component Score (PCS) and Mental Component Score (MCS) of SF-12 (12-item health survey) according to the number of attacks in the last year

ANOVA analysis to compare the results of PCS score according to the ‘number of attacks in the last year’ showed a statistically difference between patients with recurrent attacks exhibiting lower mean PCS score than patients who had only 1 attack or had no attack in the last year with a *F* value of 34.64 with p < 0.0001. Furthermore, lower MCS scores were observed in patients with recurrent attacks compared to patients that experienced just 1 attack or patients free of attack with a *F* value of 7.39 with p < 0.0001. Figure 4 shows the difference in PCS and MCS scores according to the ‘number of attacks in the last year’.

## Discussion

The porphyrias are a rare and heterogenous group of inherited disorders due to defects in the heme biosynthesis pathway that is commonly misdiagnosed due to a combination of factors like unpredictable manifestations, easily missed without a high degree of clinical suspicion, non-pathognomonic symptoms, more prevalent conditions that can explain clinical features, low prevalence and low penetrance of the genetic variants constituting a big and hard challenge for many specialists [11–13].

This work represents a large nationwide cohort of Brazilian patients with porphyria already published in the literature with a sample and importance similar to the large Norwegian Registry of Porphyria carried out by the Norwegian Center for Porphyria (NAPOS), Swedish Registry of Porphyria and the Argentine cohort of the ‘Centro de Investigaciones sobre Porfirinas y Porfirias’ (CIPYP) with the important methodological difference that this study has a cross-sectional design and does not capture retrospective or prospective data for many years like the cohorts mentioned above [14–16]. Similar to other studies, most patients in the Brazilian cohort of porphyria are female and Caucasian with a high prevalence of AIP among patients with AHP and with the difference of a greater proportion of cases with VP (10.8%) and HCP (6%) than that observed in the EXPLORE (VP = 4.4%; HCP = 2.6%) and ENVISION (VP = 2.0%; HCP = 1%) studies [5, 6].

The unemployment rate among Brazilian patients with AHP are high corresponding to 20.3% of national cohort and similar to observed in other rare disorders like Multiple Sclerosis with an unemployment rate of 40% in Poland and 19.32% in Argentina [17, 18], Fabry’s disease with a rate of 39.5% [19] and higher when compared with other disorders such as Glycogen Storage Disease type I with 9% [20], Myasthenia Gravis with 13% [21], Vasculitis with 4% [22] and lower when compared to Myotonic Dystrophy type 1 a neuromuscular disorders with systemic complications in which only one third of adult patients are employed [23].

Almost 50% of Brazilian patients with porphyria were diagnosed by a neurologist and less than 10% by a gastroenterologist or hepatologist, although the first symptom in 52% of patients was abdominal pain indicating a low suspicion of the disease among gastroenterologists and a late diagnosis only when severe neurological impairment manifest. Abdominal pain is a frequent complain in clinical practice being estimated to be 7–10% of all emergency department visits and is the most common gastrointestinal symptom in outpatient clinics and in 41% of patients the condition is labeled as nonspecific abdominal pain (NSAP) representing the most common cause of surgical intervention due to acute abdominal pain in Emergency Department [24–26]; for this reason, AHP should always be remembered as a major differential diagnosis for patients with acute or recurrent abdominal pain specially when associated with neurological features like acute weakness, sensory disturbances, autonomic failure and acute encephalopathy [3, 27].

In contrast with an American study in which most subjects (81%) reported onset of symptoms in the 2nd to 4th decades of life, more than 40% of Brazilian patients with AHP reported onset of symptoms before 18 years of age; however, with similar age at definitive diagnosis [28]. Unfortunately, the mean time to definite diagnosis in our cohort was 9.6 years like previous data published in the literature that shows a mean time of 15 years to establish the diagnosis of AHP with the need for the development of high clinical suspicion in the medical community, especially in some specific conditions such as acute flaccid paralysis, acute dysautonomia, Posterior Reversible Encephalopathy Syndrome (PRES), dark-reddish urine and hyponatremia in the context of the syndrome of inappropriate antidiuretic hormone secretion (SIADH) [2, 3, 15, 28–32]. The clinical profile during an attack of patients with AHP has some difference in relation to the EXPLORE cohort with more Brazilian patients presenting arm or leg pain 91.8% versus 77% and muscle pain 81.7% versus 50% during an attack; also, Brazilian patients exhibited a high prevalence of neuropsychiatric symptoms such as anxiety, insomnia and numbness present in more than 80% of patients and lower frequency of patients with dark urine when compared to EXPLORE cohort [6].

In relation to chronic clinical profile, more Brazilian patients reported chronic symptoms (70.9%) than EXPLORE patients (65%) with some interest differences like arm or leg pain as the major cause of pain in Brazilian patients in contrast to abdominal pain in EXPLORE cohort and a higher prevalence of anxiety, insomnia, constipation, nausea, fatigue, numbness and weakness present in more than half of patients in Brazilian patients and in a less extension with < 20% of patients in EXPLORE study; furthermore, Brazilian cohort had more patients with systemic arterial hypertension (35%) as long-term complication related to AHP than previously reported [6]. Other important clinical differences of this cohort compared to the EXPLORE study are a higher number of patients reporting zero attacks (47.2% versus 6%) and a lower proportion of patients with more than 10 attacks (4.7% versus 32%) in the last 12 months (6). Although, the frequency of Brazilian patients with recurrent attacks is high, corresponding to 25% of this cohort, in contrast to the proportion of 8% previously reported in the literature [7].

Brazilian patients with AHP have significant compromise of HRQoL in both domains (physical and mental) when compared with a normal population and the impairment of PCS (mean 39.4) are similar to the patients included on the ENVISION study (mean 38.4 on placebo group and 39.4 at Givosiran arm at baseline) [6]. Moreover, the impact of disease on mental health of Brazilian patients is severe with a high prevalence of anxiety (69.5%), as already indicated by other study in patients with Porphyria by different methods (Beck Anxiety Inventory, Beck Depression Inventory, State Trait Anxiety Disorders and Hospital Anxiety and Depression Scale) [33–36]. Of note, in our cohort the PCS and MCS have a correlation with the number of attacks in the last year with patients with recurrent attacks (> 4 attacks per year) had lower scores of HRQoL as already observed in a previous study with 81 patients with AHP 20 years ago [34, 35]. Brazilian patients with AHP have poor quality of life measured by SF-12 when compared with other rare disorders like chronic musculoskeletal disorders (PCS = 42.38; MCS = 46.57), cardiac disease (PCS = 32.5; MCS = 45.4), hemophilia (PCS = 43.69; MCS = 46.48) and inflammatory bowel disease (PCS = 36.10; MCS = 36.04) [37–40]. The medical burden of AHP disease in Brazilian patients is broad requiring another 9 years and more than 60 consultations for diagnostic definition which is higher than other rare conditions such as Hereditary Angioedema [41], Fabry Disease [42] and Pompe Disease [43] and compatible with that has been described for AHP [44]. Recently, some studies demonstrated that economic burden of AHP are high with lifetime healthcare costs for a person with AHP were estimated to be €3,030,316 due to frequent hospitalizations associated with porphyria attacks compared to the general population and that elimination of AHP attacks could also lead to reductions in disability payments of €179,184 and healthcare cost savings of €1,511,027 per patient [45] which is important on Brazilian context when 41.8% of patients with AHP depends exclusively of public health system and 24.5% of the patients were treated with heme arginate in more than 6 attacks throughout the history of disease.

## Conclusions

Our study adds new knowledge on the type and prevalence of chronic manifestations related to AHP and new data about the natural history and burden of disease for these rare inherited disorders due to a liver dysfunction that makes an important different diagnosis for many medical specialties.

Additionally, this national registry reinforces the current knowledge that AHP should be considered a chronic condition complicated by acute attacks with frequent and disabling progressive chronic symptoms present in more than 65% of the patients, most of them related to neurological and psychiatric impairment.

Moreover, our study fortifies the knowledge that patients with AHP with recurrent attacks had a poor quality of life and great disability representing an important public health problem that can be change with actions for disease awareness and medical education driven by the advent of new innovative genetic therapies based on interference RNA technology mainly represented by Givosiran.

## Data Availability

The datasets are not publicly available due to individual privacy reasons (patients’ confidentiality).

## Abbreviations

AHP: Acute hepatic porphyrias
AIP: Acute intermittent porphyria
ALA: Delta-aminolevulinic acid
ALAD: Delta-aminolevulinate dehydratase
ALAS: 5-Aminolaevulinic acid synthase
BMI: Body mass index
BP: Bodily pain
CIPYP: Centro de Investigaciones sobre Porfirinas y Porfirias
VP: Variegate porphyria
EP: Erythropoietic porphyria
GH: General health
HCP: Hereditary coproporphyria
HRQoL: Health-related quality of life
SF-12v2: SF-12 health survey version 2
IQR: Interquartile range
MCS: Mental component summary
MH: Mental health
NAPOS: Norwegian registry of porphyria
NSAP: Nonspecific abdominal pain
PBG: Porphobilinogen
PCS: Physical component summary
PF: Physical functioning
PRES: Posterior reversible encephalopathy syndrome
RE: Role-emotional
REDcap: Research electronic data capture
RP: Role physical
SD: Standard deviation
SF: Social functioning
SIADH: Syndrome of inappropriate antidiuretic hormone secretion
siRNA: Small interfering RNA
VT: Vitality

## References

[1] Layer G, Reichelt J (2010). Structure and function of enzymes in heme biosynthesis. Protein Sci.

[2] Puy H, Gouya L (2010). Porphyrias. Lancet.

[3] Souza PVS, Badia BML (2021). Acute hepatic porphyrias for the neurologist: current concepts and perspectives. Arq Neuropsiquiatr.

[4] Bissel DM, Anderson KE (2017). Porphyria. N Engl J Med.

[5] Balwani M, Sardh E, Ventura P (2020). Phase 3 trial of RNAi therapeutic givosiran for acute intermittent porphyria. N Engl J Med.

[6] Gouya L, Ventura P, Balwani M (2020). EXPLORE: a prospective, multinational, natural history study of patients with acute hepatic porphyria with recurrent attacks. Hepatology.

[7] Schmitt C, Lenglet H, Yu A (2018). Recurrent attacks of acute hepatic porphyria: major role of the chronic inflammatory response in the liver. J Intern Med.

[8] Maruish ME (2012). User’s manual for the SF-12v2 health survey.

[9] Andersen J, Thomsem J (2020). Health-related quality of life in porphyria cutanea tarda: a cross-sectional registry based study. Health Qual Life Outcomes.

[10] Harris PA, Taylor R, Minor BL (2019). The REDCap consortium: building an international community of software platform partners. J Biomed Inform.

[11] Barletta E, Belsuzarri TA (2021). Acute neurological manifestations of porphyrias and its types: a systematic-review. Cardiovasc Hematol Agents Med Chem.

[12] O’Malley R, Rao G (2018). Porphyria: often discussed but too often missed. Pract Neurol.

[13] Marcacci M, Ricci A (2022). Challenges in diagnosis and management of acute hepatic porphyrias: from an uncommon pediatric onset to innovative treatments and perspectives. Orphanet J Rare Dis.

[14] Bylesjö I, Wikberg A (2009). Clinical aspects of acute intermittent porphyria in northern Sweden: a population-based study. Scan J Clin Lab Invest.

[15] Thunell S, Floderus Y (2006). Porphyria in Sweden. Physiol Res.

[16] Martinez MDC, Cerbino G (2021). Clinical, biochemical, and genetic characterization of acute hepatic porphyrias in a cohort of Argentine patients. Mol Genet Genom Med.

[17] Koziarska D, Krol J (2018). Prevalence and factor leading to unemployment in MS (multiple sclerosis) patients undergoing immunomodulatory treatment in Poland. PLoS ONE.

[18] Vanotti S, Eizaguirre MB, Ciufia NP (2021). Employment status monitoring in an Argentinian population of patients with multiple sclerosis: particularities of a developing country. Work.

[19] Körver S, Geurtsen G, Hollak CEM (2020). Depressive symptoms in Fabry disease: the importance of coping, subjective health perception and pai. Orphanet J Rare Dis.

[20] Garbade SF, Ederer V, Burgard P (2021). Impact of glycogen storage disease type I on adult daily life: a survey. Orphanet J Rare Dis.

[21] Ignatova V, Kostadinov K, Vassileva E (2022). Socio-Economic Burden of Myasthenia gravis: a cost-of-illness study in Bulgaria. Front Public Health.

[22] Sreih AG, Cronin K, Shaw DG (2021). Diagnostic delays in vasculitis and factors associated with time to diagnosis. Orphanet J Rare Dis.

[23] De Antonio M, Dogan C, Daidj F (2019). The DM-scope registry: a rare disease innovative framework bridging the gap between research and medical care. Orphanet J Rare Dis.

[24] Cervellin G, Mora R, Ticinesi A (2016). Epidemiology and outcomes of acute abdominal pain in a large urban Emergency Department: retrospective analysis of 5,340 cases. Ann Transl Med.

[25] Hastings RS, Powers RD (2011). Abdominal pain in the ED: a 35 year retrospective. Am J Emerg Med.

[26] Fagerström A, Paajanen P, Saarelainen H (2017). Non-specific abdominal pain remains as the most common reason for acute abdomen: 26-year retrospective audit in one emergency unit. Scan J Gastroenterol.

[27] De Souza PVS, Badia BML (2021). Acute hepatic porphyria: pathophysiological basis of neuromuscular manifestations. Front Neurosci.

[28] Bonkovsky HL, Maddukuri VC, Yazici C (2014). Acute porphyrias in the USA: features of 108 subjects from porphyrias consortium. Am J Med.

[29] Kondo M, Yano Y (2004). Porphyrias in Japan: compilation off all cases reported through 2002. Int J Hematol.

[30] Alfadhel M, Saleh N (2014). Acute intermittent porphyria caused by novel mutation in HMBS gene, misdiagnosed as cholecystitis. Neuropsychiatr Dis Treat.

[31] Jaramillo-Calle DA, Solano JM (2019). Porphyria-induced posterior reversible encephalopathy syndrome and central nervous system dysfunction. Mol Genet Metab.

[32] Solares I, Tejedor M, Jericó D (2020). Management of hyponatremia associated with acute porphyria-proposal for the use of tolvaptan. Ann Transl Med.

[33] Millward LM, Kelly P (2005). Anxiety and depression in the acute porphyrias. J Inherit Metab Dis.

[34] Francesca G, Nicolli A (2022). Psychological aspect and quality of life in Porphyrias: a review. Diagnostics (Basel).

[35] Millward LM, Kelly P (2001). Self-rated psychosocial consequences and quality of life in the acute hepatic porphyrias. J Inherit Metab Dis.

[36] Naik H, Stoecker M (2016). Experiences and concerns of patients with recurrent attacks of acute hepatic porphyria: a qualitative study. Mol Genet Metab.

[37] Cuesta-Vargas AI, González-Sanchez M (2013). Effect on health-related quality of life of a multimodal physiotherapy program in patients with chronic musculoskeletal disorders. Health Qual Life Outcomes.

[38] Bahall M, Legall G (2020). Quality of life among patients with cardiac disease: the impact of comorbid depression. Health Qual Life Outcomes.

[39] Shah RM, Banahan BF, Holmes ER (2018). An evaluation of the psychometric properties of the sf-12v2 health survey among adults with hemophilia. Health Qual Life Outcomes.

[40] Chiarini M, Di Simone E, Scafuro C (2017). Health self-perception in patient with Crohn’s disease: a web survey. Clin Ter.

[41] Isono M, Kokado M (2022). Why does it take so long for rare disease patients to get an accurate diagnosis?—a qualitative investigation of patient experiences of hereditary angioedema. PLoS ONE.

[42] Martins AM, Cabera G, Molt F (2019). The clinical profiles of female patients with Fabry disease in Latin America: a Fabry Registry analysis of natural history data from 169 patients based on enzyme replacement therapy status. JIMD Rep.

[43] Reuser AJJ, van der Ploeg AT, Chien YH (2019). GAA variants and phenotypes among 1,079 patients with Pompe disease: data from the Pompe Registry. Hum Mutat.

[44] Neeleman RA, Wagenmakers MAEM, Koole-Lesuis RH (2018). Medical and financial burden of acute intermittent porphyria. J Inherit Metab Dis.

[45] Connolly MP, Kotsopoulos N (2021). Estimating the broader fiscal consequences of acute hepatic porphyria (AHP) with recurrent attacks in Belgium using a public economic analytic framework. Orphanet J Rare Dis.

